# HXMS: a standardized file format for HX/MS data

**DOI:** 10.1101/2025.10.14.682397

**Published:** 2025-10-16

**Authors:** Kyle C. Weber, Chenlin Lu, Roberto Vera Alvarez, Bruce D. Pascal, Anum Glasgow

**Affiliations:** 1 Department of Biochemistry and Molecular Biophysics, Columbia University, New York, NY 10032, USA.; 2 Omics Informatics LLC, Honolulu, HI 96813, USA

## Abstract

**Motivation:**

Hydrogen-deuterium exchange/mass spectrometry (HX/MS) is a rapidly expanding technique used to investigate protein conformational ensembles. The growing popularity and utility of HX/MS has driven the development of diverse instrumentation and software, resulting in inconsistent, non-standardized data analysis and representation. Most HX/MS data formats also employ only centroid-level representations of the data rather than full isotopic mass spectra, reducing the information content of the data and limiting downstream quantitative analysis.

**Results:**

Inspired by reliable protein structure and genomics data formats, we present HXMS, a unified, lightweight, scalable, and human-readable file format for HX/MS data. The HXMS format preserves the isotopic mass envelopes for all peptides, captures the full experimental time-course including the fully deuterated control samples, and contains all other key information. It supports multimodal distributions, post-translational modifications (PTMs), and experimental replicates. To promote compatibility with existing HX/MS workflows, we also developed PFLink, a Python package that converts exported data files from commonly used HX/MS analysis software packages to the HXMS format. PFLink and the HXMS format will enable more quantitative, higher-resolution data processing, improved data sharing and storage among HX/MS practitioners, future machine learning applications, and further developments in HX/MS analysis.

**Availability and implementation:**

PFLink is publicly available to install locally on HuggingFace, alongside documentation, or use online at HuggingFace (https://huggingface.co/spaces/glasgow-lab/PFLink). We also included a generic unfilled PFlink custom CSV file that users may populate with key experimental conditions and results, which can then be read and converted into the HXMS format.

## 1 Introduction

Hydrogen-deuterium exchange/mass spectrometry (HX/MS) is a powerful biophysical method for probing protein folding and conformational ensembles (Englander et al., 2016). HX/MS measures the rate at which backbone amide hydrogens in a protein are replaced with deuterium atoms when exposed to deuterated buffers, providing time-resolved insights into protein structure and conformational stability. The commercial availability of automated systems to perform HX/MS experiments (Anacleto et al., 2023; Kish et al., 2023) and analyze HX/MS data has increased its appeal for studying large, complex proteins, allowing scientists to probe many pharmacological targets for applications such as drug discovery, epitope mapping, and protein characterization (Hamuro, 2017; Glasgow et al., 2023; Wells et al., 2025; Wales et al., 2024; Gramlich et al., 2021; Wang et al., 2012; Gertsman et al., 2009; Shaw et al., 2023).

Despite the widespread adoption of HX/MS in the structural biology community, most practitioners analyze HX/MS data at the peptide level using the centroids of the isotopic mass envelopes. The practice of using MS centroids rather than the full MS envelopes limits opportunities for quantitative treatment of HX/MS data due to the resulting information loss and degeneracy (Kan et al., 2019; Lu et al., 2025). This problem is made worse by the large size and instrumentation-specific formats of the raw MS data files, and the varied export formats of available HX/MS software. Altogether, sharing HX/MS data is difficult and cumbersome since no suitable standardized format exists.

**Figure 1.**
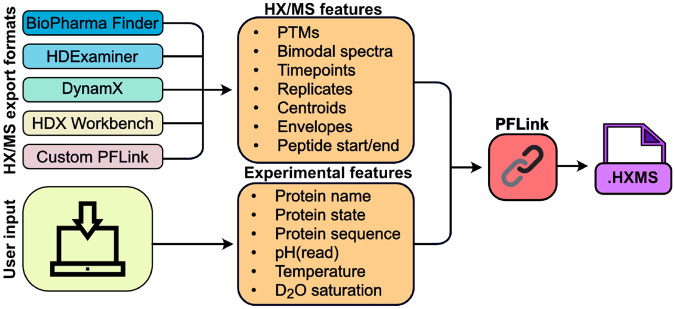
HXMS file creation using PFLink. The user provides two types of information to PFLink: 1) HX/MS data exported directly from the analysis software, or in the PFLink custom format, and 2) details about the protein and experimental conditions. PFLink automatically compiles this information in the standardized HXMS format.

Here we present HXMS, a lightweight and human-readable file format for HX/MS data that includes the experimental isotopic mass envelopes and all necessary information for high-resolution data analysis. We also introduce PFLink, a software package that converts HX/MS data files produced by four different HX/MS analysis programs (or our custom data file) to the HXMS format (Fig. 1). The standardized HXMS format will advance data processing, data sharing, and technical developments within and beyond the HX/MS community.

## 2 Results

### 2.1 Metadata section format

The HXMS file format consists of three main sections: a metadata section, an experimental data section, and a post-translational modification (PTM) dictionary section. The metadata section describes the required experimental conditions used in the HX/MS experiment (Table 1). Important variables include the protein name, sequence, and state; the temperature, the pH(READ), and the D_2_O saturation. The user may add additional metadata using a “REMARK” header in the metadata section. Metadata objects must start with either "METADATA" or "REMARK", followed by a tab. The title comes next, followed by a final tab before the data or remark itself, and ends with a new line character.

### 2.2 Experimental data section format

The experimental data section contains all information necessary to represent a singular timepoint in any HX/MS experiment (Table 2). The "TP" tag is used to declare a timepoint, followed by a peptide timepoint index, “INDEX”, which incrementally increases by 1. If any timepoint represents a peptide that exhibits a multimodal distribution, the "MOD" column denotes the distribution using alphabetical indices (*e.g.*, "A" is the default for a singular distribution, but “B” can be used for a peptide with an additional mode). The "START" and "END" columns indicate the peptide's beginning and end indices. These are inclusive of both ends, and indexing starts at 1 for the first amino acid in the protein. If there are multiple experimental measurements of the same peptide, the "REP" column may be indexed to denote this, where index 0 is the default for a single replicate. Replicates should be incremented only for new experimental measurements, not for timepoints in the same experiment. The "PTM_ID" column catalogs PTMs. For each unique PTM, whether it is on the same peptide or a different one, the counter is incremented. The default setting for no PTM uses the index 0000. The "PTM_ID" is used in the lookup table in the PTM section of the HXMS file to provide information about each modification, as described further in Section 2.3. The "TIME(SEC)" column indicates the duration of the sample incubation in D_2_O before it was quenched. Time is reported in scientific notation, except for fully deuterated samples, which are reported as “inf”. The "UPTAKE" column denotes the amount of deuterium incorporated. This is calculated by subtracting the centroid of the 0 s timepoint from the centroid for each given timepoint, for each peptide and replicate. Finally, the "ENVELOPE" column contains full isotopic mass envelopes for non-centroid data. There is no limit to the number of peaks that can be included. The peaks are separated by commas and are normalized to sum to 1.

### 2.3 PTM section format

The PTM section serves as a dictionary, providing detailed information for any PTMs in the dataset (Table 3). This section links the ‘PTM_ID’ from the experimental data section to a comprehensive description of the modification. The ‘PTM’ tag is used to declare a PTM, followed by the ‘PTM_ID’ in the timepoint series section. The description of the entry is in the ‘CONTENT’ column, which describes the modified amino acid within the protein sequence. If there are multiple PTMs in one peptide, one may place a comma and denote the next PTM on the same line. We recommend using PDB ligand ID for simplicity and clarity, but any declaration format can be used due to the high diversity of PTMs.

### 2.4 PFLink: software to convert HX/MS files to the HXMS format

To enable widespread compatibility and accessibility with the HXMS format, we developed PFLink, a Python package to convert exported HX/MS data files from several widely used commercial and academic HX/MS data analysis programs. PFLink is compatible with exported data from BioPharma Finder (Fisher), HDExaminer (Trajan), DynamX (Waters), and HDX Workbench (Pascal et al., 2012) (Fig. 1, Supplemental Data 1). As most HX/MS analysis software packages currently only support the export of HX/MS data in the centroid format, PFLink can write HXMS files originating from any of these programs in the centroid format.

BioPharma Finder and DynamX report deuterium uptake and modifications directly, so HX/MS data exported from these programs can be used as-is. In contrast, PFLink recalculates deuterium uptake as reported by HDX Workbench by first identifying the zero timepoint for each peptide (or an average of the replicates, if any replicate is missing a zero timepoint), and then normalizes each timepoint by subtracting this zero timepoint value. PTMs are supported on all file formats except for HDExaminer and BioPharma Finder; for these programs, they must be added to the HXMS file manually.

Because both HDX Workbench and HDExaminer support exporting complete isotopic mass spectra for all peptides, PFLink can also generate HXMS files in the full-spectrum format when provided with this input. Alternatively, users may choose to run PFLink using the custom data format supplied in Supplemental Data 1. The resulting HXMS files are compatible with the quantitative HX/MS analysis methods PFNet and FEATHER (Lu et al., 2025).

### 2.5 Two examples

We include two sets of HXMS files on two proteins as examples (Supplemental Data 2): one set for *E. coli* DHFR in its apo state and two inhibitor-bound states (Lu et al., 2025), and another set for the pre- and post-fusion stabilized states of the herpes simplex virus (HSV) glycoprotein B (gB) (Roark et al., 2025). In both cases, we collected the data using a Bruker MaXis II LC-QTOF mass spectrometer. Both datasets were processed using PIGEON-derived peptide lists (Lu et al., 2025) in HDExaminer. State-specific HXMS files were then generated using PFLink: apo, methotrexate (MTX)-bound, and trimethoprim (TMP)-bound for DHFR; and pre- and post-fusion for HSV gB. The HSV gB HXMS files contain bimodal spectra. Examples of PTMs can be found in the DynamX supporting files (Supplemental Data 1).

## 3 Discussion

In this article, we introduced HXMS and PFLink: a standardized, lightweight, and human-readable HX/MS data format, and a software package to convert HX/MS data to this format. In designing the HXMS format, we drew from the most successful elements of protein structure and genomics data formats to establish a flexible framework for HX/MS data exchange. The key advantage of the HXMS data format is its inclusion of isotopic mass envelopes and all other data necessary for quantitative and high-resolution data analysis. PFLink preserves all features of the data upon conversion to HXMS: experimental and biological replicates; PTMs; multimodal distributions; all measurements, including fully deuterated samples; and, when necessary or preferable, a centroid-level data representation.

While other HX/MS data formats are widely in use, these can often only be used with specific mass spectrometers and rely on centroid-level representations, which fail to capture the full complexity of HX/MS data. The lack of standardization in the field, combined with the large size of raw HX/MS data files, limits the extraction of valuable information from past HX/MS studies. By contrast, the HXMS format is compatible with major commercial and academic HX/MS analysis software packages that enable accurate and quantitative determination of high-resolution ensemble energies from HX/MS data (Lu et al., 2025). The HXMS format will improve data sharing and storage for the HX/MS community and enable future machine learning applications that require large amounts of HX/MS data from many practitioners.

## Supplementary Material

Supplement 1

## Figures and Tables

**Table 1 –:** Metadata information and requirements.

Metadata	Information	Example	Required
PROTEIN_SEQUENCE	Protein sequence	GSHMKTVEVNGADASDDN	Yes
PROTEIN_NAME	Name of the protein	Human PFK-1	No
PROTEIN_STATE	State of the protein	APO	No
TEMPERATURE (K)	Temperature in Kelvin	293.15	Yes
pH(READ)	pH(read)	6.0	Yes
D2O_SATURATION	D_2_O saturation	0.91	Yes

**Table 2 –:** HX/MS data

Header name	Information	Number of characters	Separation character	Example
TITLE	Type of data	12	N/A	TP
INDEX	Peptide index	8	N/A	0
MOD	Bimodal index	7	N/A	A
START	Peptide start position	7	N/A	1
END	Peptide end position	7	N/A	10
REP	Experiment number	5	N/A	0
PTM_ID	PTM ID used for the PTM lookup table	8	N/A	0000
TIME(SEC)	Incubation time in D_2_O	16	N/A	0.000000e+00
UPTAKE	Centroid uptake relative to 0 time point	9	N/A	0.00
ENVELOPE	Mass spec full envelope	inf, 4 per peak	,	0.527,0.298,0.116,0.036,0.000,0.023,0.000

**Table 3 –:** PTM format

Header name	Information	Number of characters	Separation character	Example
TITLE_PTM	Type of data	12	N/A	PTM
PTM_ID	PTM ID used for the PTM lookup table	8	N/A	0000
CONTENT	The PTM denoted by the absolute position of the PTM in the peptide and PTM ID	inf	,	Phosphoryl STY (18)
